# Supplementary material for: Alveolar macrophages are critical for broadly-reactive antibody-mediated protection against influenza A virus in mice
Source: Nat Commun. 2017 Oct 10;8:846. doi: 10.1038/s41467-017-00928-3 (PMC5635038; doi:10.1038/s41467-017-00928-3)
Supplement: Supplementary file 2 — Supplementary Information [file 41467_2017_928_MOESM2_ESM.pdf]

A

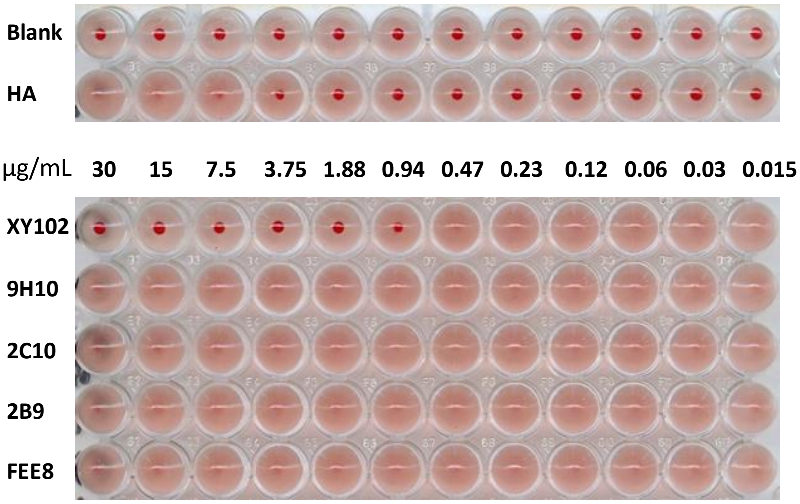

Supplementary Figure 1. *In vitro* characterizations of mAb 2B9, 2C10 and FEE8. (A) Sixteen chicken hemagglutination units (4 wells) of X31 virus was pre-incubated with each antibody with a starting concentration of 30 µg per mL before addition of 50 µL of 0.5% chicken red blood cells. PBS only (Blank) was used as a negative control. MAb XY102 (HAI+) served as a positive control.

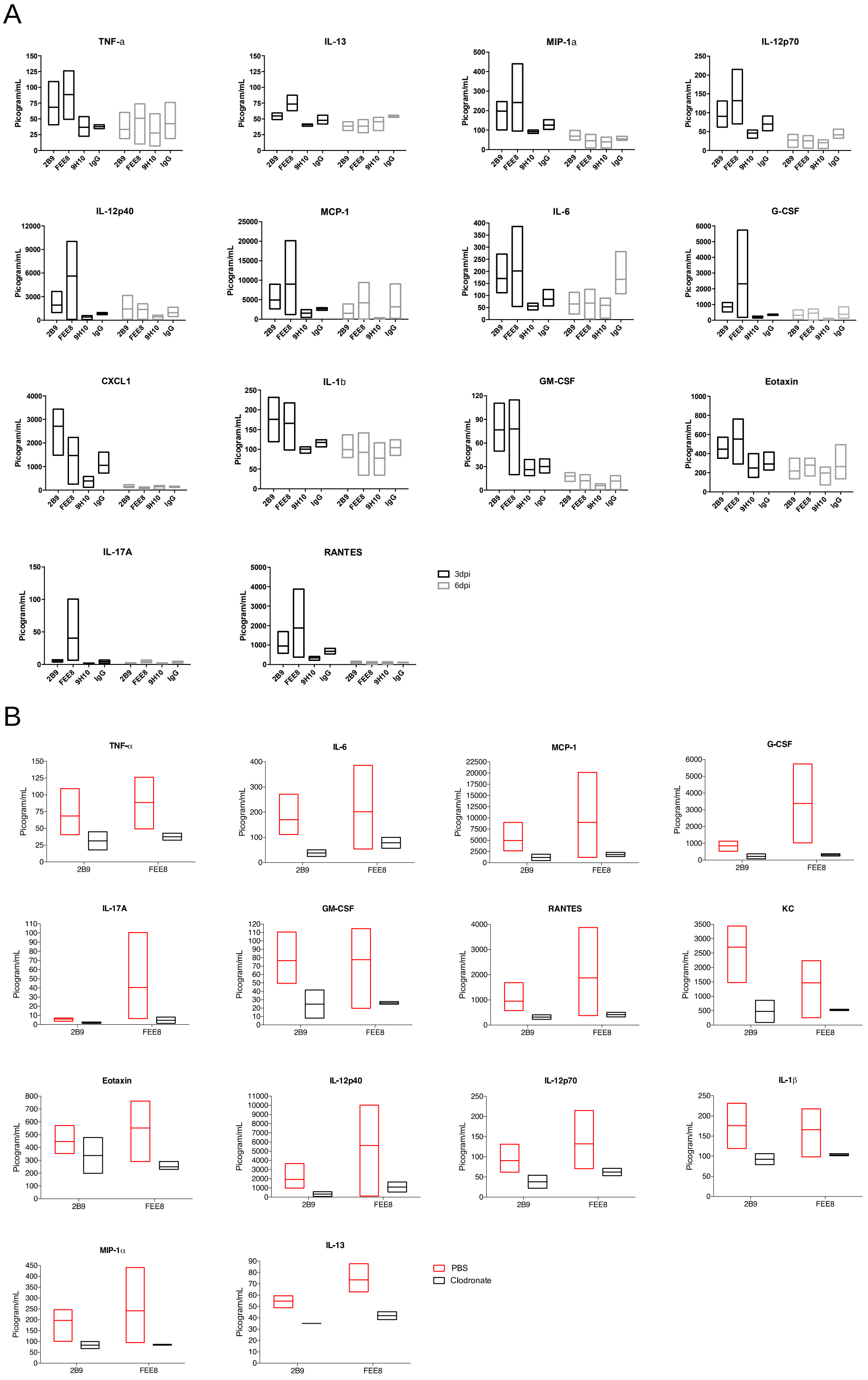

Supplementary Figure 2. Protein levels of differentially induced cytokines and chemokines in BALF of nonNAb-treated mice. BALB/c mice were administered IP with 15 mg per kg of 2B9 or FEE8 and then challenged with a lethal dose of X31 (5 mLD_{50}). (A) At 3 and 6 dpi, BALF were harvested from the mice and analyzed using a multiplex bead array assay for cytokine/chemokine production. (B) Additional mice that received 2B9 or FEE8 were administered with clodronate to deplete AMφ. At 3 dpi, BALF were harvested from these mice and their cytokine levels were compared with that of non-depleted groups. Values represent mean ± SD (n=3 mice

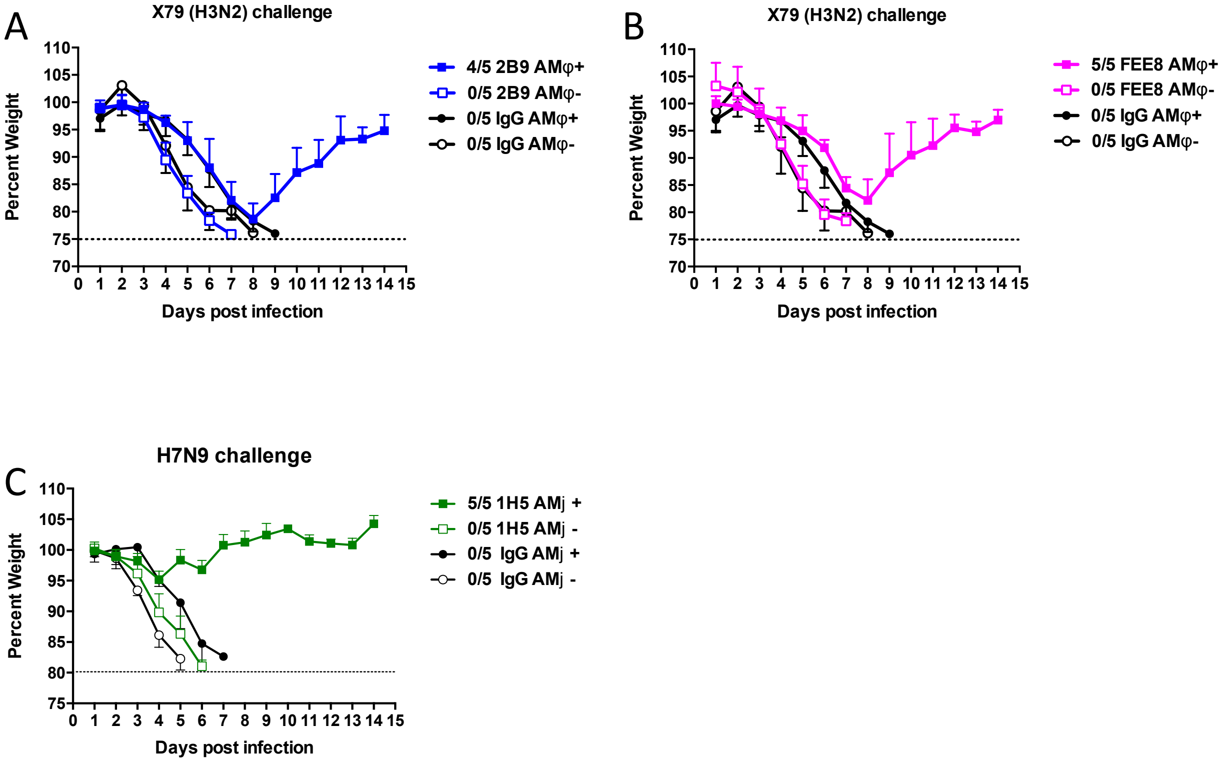

Supplementary Figure 3. Heterologous protection provided by broadly-reactive nonNAbs requires AMφ. BALB/c mice were depleted of AMφ at -2 and 0 (day of challenge) using clodronate-liposome administered IN. Control mice received PBS-liposome. Mice were treated IP with 15 mg per kg (A) 2B9, (B) FEE8, or (C) 1H5 before the challenge with 5 mLD_{50} of X79 (a reassortant virus with the HA and NA of A/Philippines/82 and the internal proteins of PR/8) or 10 mLD_{50} of A/Shanghai/1/13 (H7N9). A mAb against GST was used as an IgG control. The ratios in the figure legends indicate the number of animals that survived challenge over total number of animals per group. Values represent mean ± SD (n=5 mice per group).

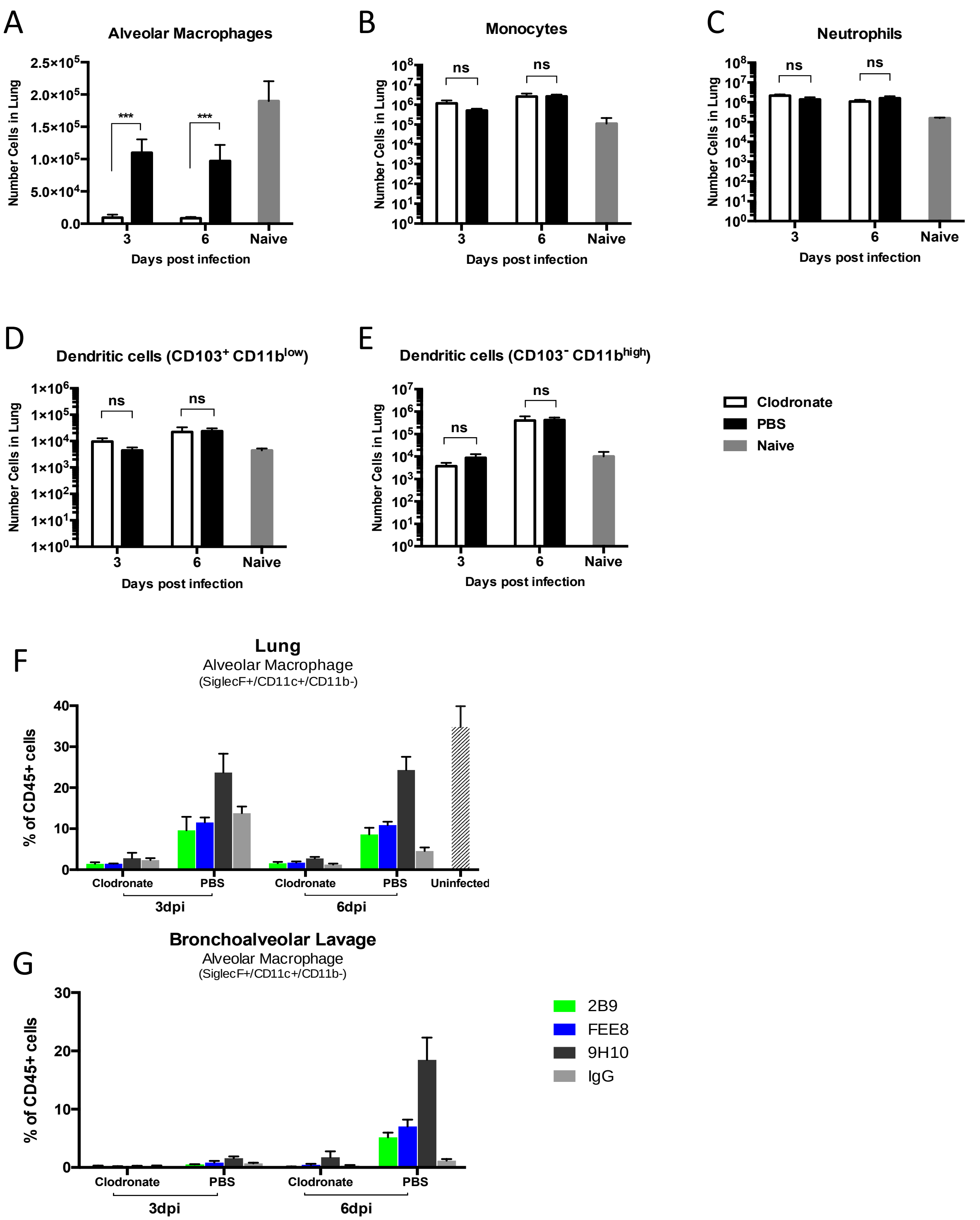

Supplementary Figure 4. Clodronate treatment results in depletion of AMφ without affecting other innate immune cell populations. BALB/c mice were treated on day -2 and 0 with clodronate- or PBS-liposomes delivered IN. On day 0, these mice were challenged with a lethal dose of X31. At 3 and 6 dpi, lung tissues were collected for immune cell phenotyping by flow cytometry. Total numbers of cells in lung: (A) AMφ [SiglecF+/CD11c+/CD11b-], (B) monocytes [Ly6G-/Ly6C+/CD11b+], (C) neutrophils [Ly6G+/Ly6C+/CD11b+], (D, E) dendritic cells [(D) CD45+/CD11c+/MHCII+/CD103+/$CD11b^{low}$, (E) CD45+/CD11c+/MHCII+/CD103-/$CD11b^{high}$]. (F, G) Percentages represent the number of AMφ over total CD45+ cells in the (F) lung and (G) BALF for mice that received mAb 2B9, FEE8 or 9H10 (15 mg per kg). Two-way ANOVA and Sidak's multiple comparisons tests were used to determine statistical significance (GraphPad Prism). Error bars represent SD (n=3 per group). For all panels: $*P \leq 0.05$, $**P \leq 0.01$, $***P \leq 0.001$; ns, not significant.

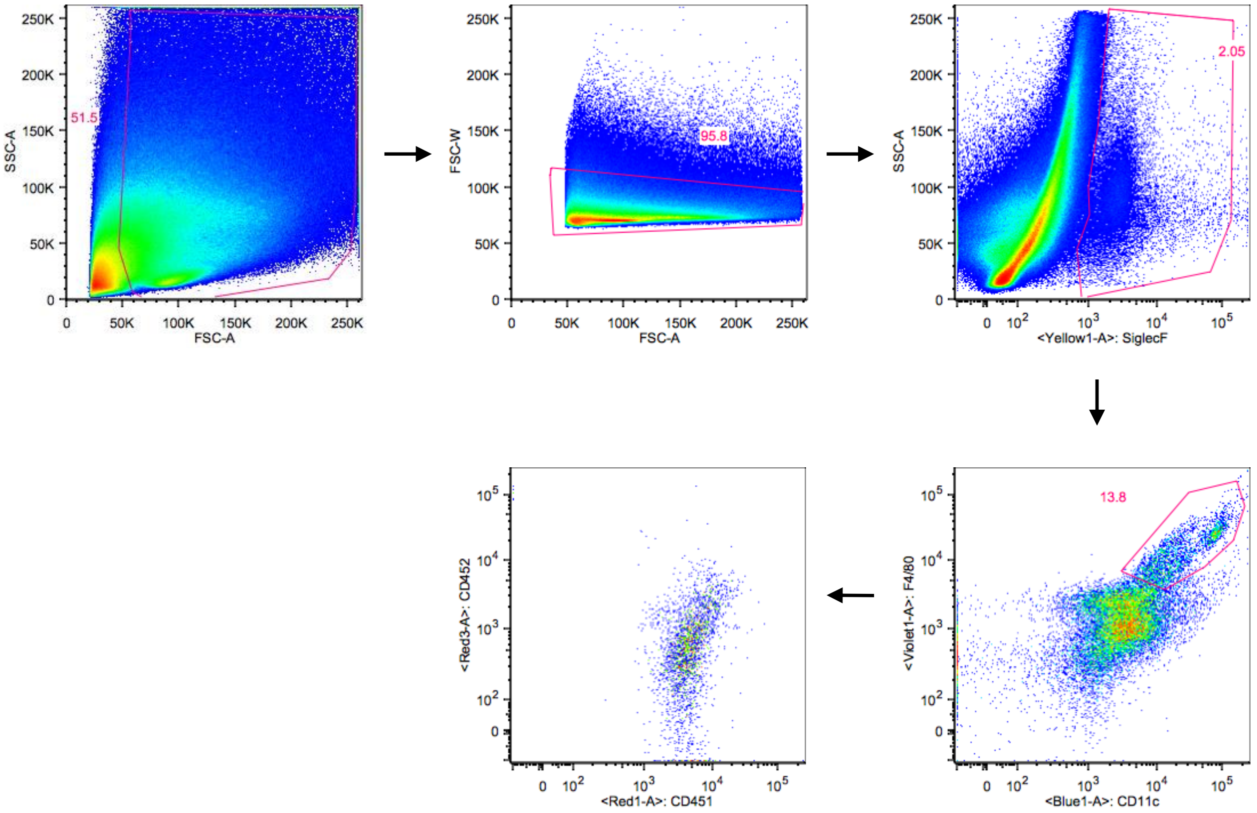

Supplementary Figure 5. Reconstitution of AMφ in recipient mice. Recipient GM-CSF knockout (CD45.2) mice were adoptively transferred (intranasally) with AMφ isolated from donor female B6.SJL (CD45.1) mice. Three days post adoptive transfer, a recipient female GM-CSF knockout (CD45.2) mice was sacrificed, lung harvested and donor AMφ (CD45.1) were identified by flow cytometry.

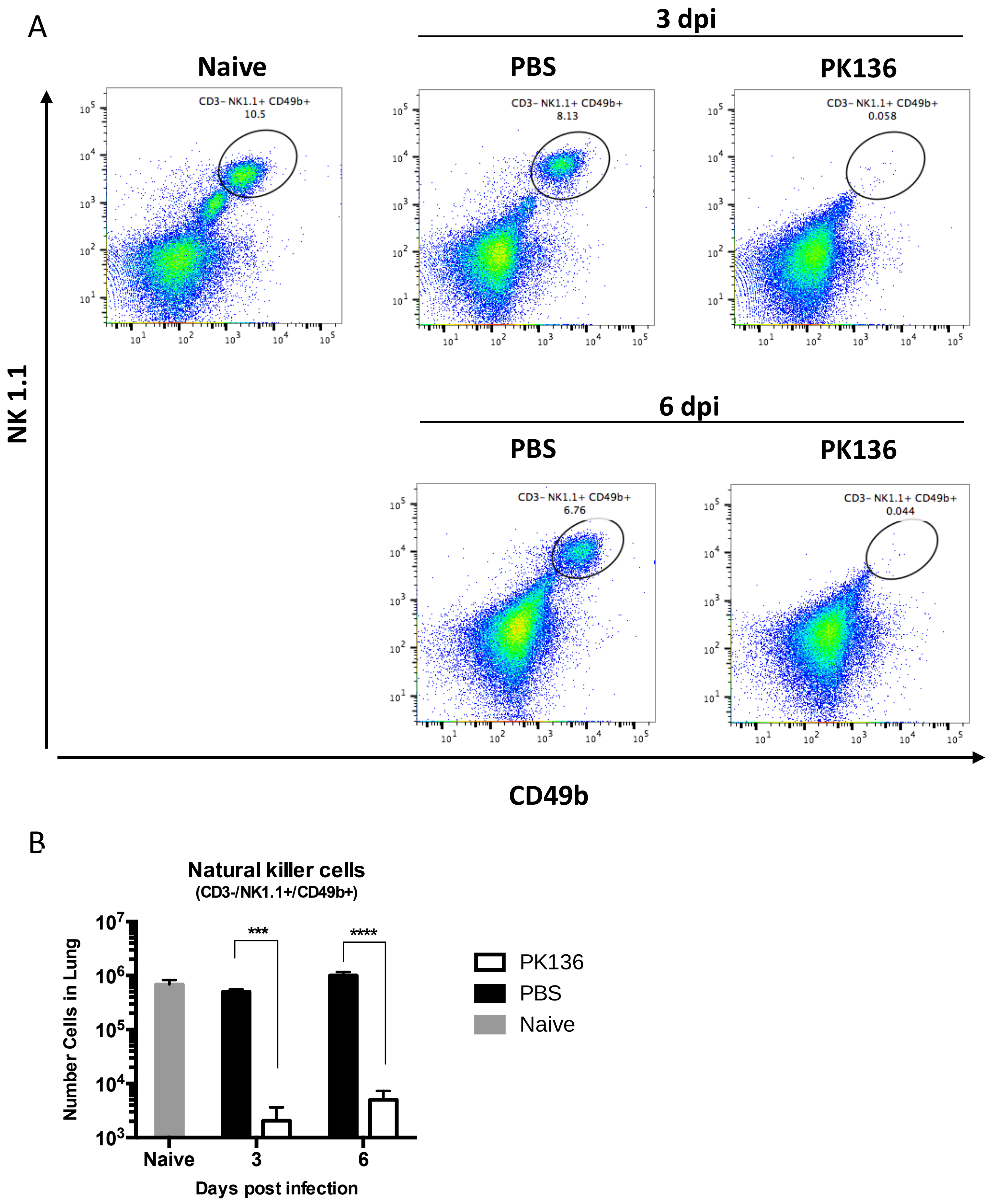

Supplementary Figure 6. Administration of mAb PK136 in C57/BL6J mice depleted NK cells in the lung. C57/BL6J mice were injected IP with 250 µg PK136 on day -2, 0 and 5. On day 0, these mice were infected with a lethal dose of X31. At 3 and 6 dpi, lung tissues were collected for immune cell phenotyping by flow cytometry. (A) Gates were set on the CD3-/NK 1.1+/CD49b+ subset. (B) Total numbers of cells in lung. Two-way ANOVA and Sidak's multiple comparisons tests were used to determine statistical significance (GraphPad Prism). For all panels: *P ≤ 0.05, **P ≤ 0.01, ***P ≤ 0.001; ns, not significant.

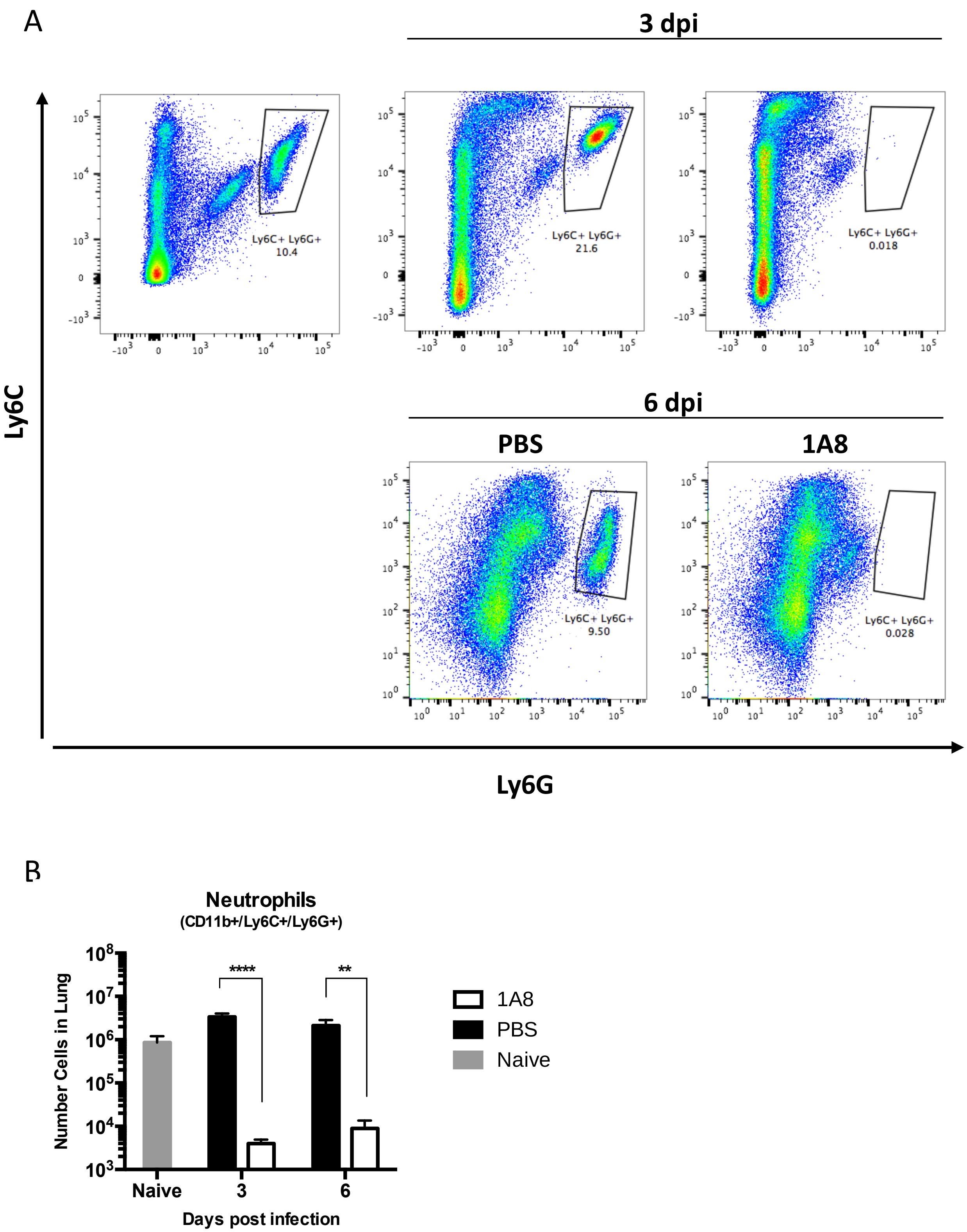

Supplementary Figure 7. Administration of mAb 1A8 in BALB/c mice depleted neutrophils in the lung. BALB/c mice were injected IP with 250 µg 1A8 on day -1, 1, 3 and 5. On day 0, these mice were infected with lethal dose of X31. At 3 and 6 dpi, lung tissues were collected for immune cell phenotyping by flow cytometry. (A) Gates were set on the CD11b+/Ly6C+/Ly6G+ subset. (B) Total numbers of cells in lung. Two-way ANOVA and Sidak's multiple comparisons tests were used to determine statistical significance (GraphPad Prism). For all panels: *P ≤ 0.05, **P ≤ 0.01, ***P ≤ 0.001; ns, not significant.

Supplementary Table 1. Binding of monoclonal antibodies by ELISA

Subtype	Isolate	9H10	2B9	2C10	FEE8
		EC$_{50}$(μg·mL^{-1})			
H3N2	A/HongKong/1/1968	0.009	0.042	0.015	0.057
H3N2	A/Victoria/3/1975	0.020	0.040	0.059	0.105
H3N2	A/Philippines/2/1982	0.003	22.09	13.18	0.005
H4N6	A/duck/Czechoslovakia/1956	>100	>100	>100	>100
H7N9	A/Shanghai/02/2013	>100	>100	>100	>100
H10N7	A/mallard/Interior Alaska/10BM01929/2010	0.012	>100	>100	>100
